# CB_1_ Activity Drives the Selection of Navigational Strategies: A Behavioral and c-Fos Immunoreactivity Study

**DOI:** 10.3390/ijms21031072

**Published:** 2020-02-06

**Authors:** Daniela Laricchiuta, Francesca Balsamo, Carlo Fabrizio, Anna Panuccio, Andrea Termine, Laura Petrosini

**Affiliations:** Laboratory of Experimental and Behavioral Neurophysiology, Fondazione Santa Lucia, 00143 Rome, Italy; francesca.balsamo93@gmail.com (F.B.); carlo.fabrizio217@gmail.com (C.F.); anna.panuccio@gmail.com (A.P.); andreatermine1@hotmail.it (A.T.); laura.petrosini@uniroma1.it (L.P.)

**Keywords:** endocannabinoid system, spatial learning, hippocampus, dorsal striatum, amygdala, Circular Hole Board, AM251, mice

## Abstract

To promote efficient explorative behaviors, subjects adaptively select spatial navigational strategies based on landmarks or a cognitive map. The hippocampus works alone or in conjunction with the dorsal striatum, both representing the neuronal underpinnings of the navigational strategies organized on the basis of different systems of spatial coordinate integration. The high expression of cannabinoid type 1 (CB_1_) receptors in structures related to spatial learning—such as the hippocampus, dorsal striatum and amygdala—renders the endocannabinoid system a critical target to study the balance between landmark- and cognitive map-based navigational strategies. In the present study, mice treated with the CB_1_-inverse agonist/antagonist AM251 or vehicle were trained on a Circular Hole Board, a task that could be solved through either navigational strategy. At the end of the behavioral testing, c-Fos immunoreactivity was evaluated in specific nuclei of the hippocampus, dorsal striatum and amygdala. AM251 treatment impaired spatial learning and modified the pattern of the performed navigational strategies as well as the c-Fos immunoreactivity in the hippocampus, dorsal striatum and amygdala. The present findings shed light on the involvement of CB_1_ receptors as part of the selection system of the navigational strategies implemented to efficiently solve the spatial problem.

## 1. Introduction

Spatial navigation is a complex ability based on the integration of sensory-motor, perceptual, cognitive and emotional processes [1,2]. Subjects show a great individual variability in navigational strategies they carried out, because of external factors, such as landmark differentiation, visual access to environmental cues, complexity of spatial layout, as well as of internal factors, such as biological substrates, individual differences and spatial cognitive style [3]. The navigational strategies are organized on the basis of different systems of spatial coordinate integration. Namely, the navigational strategies related to landmark are based on representations encompassing visual information or connecting landmarks and paths through an egocentric (body-centered) system of reference [4,5]. Thus, the navigational strategies based on landmark are supported by associative learning involving responses to visually salient cues, without providing any spatial information [6]. These strategies are habitual and almost automatic [7,8], cognitively less demanding and scarcely flexible [9].

Conversely, the navigational strategies based on a global representation of the environment mainly involve an allocentric (world-centered) system of reference. Such a system allows moving into the space to reach a goal by referring to an internalized cognitive map, in which the location of an object is represented in spatial relationships with the other objects of the environment [10]. The allocentric navigational strategies involve learning events and environmental relations, integrating them in a spatial cognitive map, remembering the map and then deploying it to plan trajectories and paths [11]. Thus, they are cognitive tasks highly demanding and flexible. Notably, to promote efficient explorative behaviors the subject uses Landmark-based Navigational Strategy (L-NS) or Cognitive Map-based Navigational Strategy (CM-NS) according to specific factors [12]. For example, emotional and stressful factors or pharmacological treatments tend to increase the implicit processes linked to habits, and to be detrimental on allocentric navigational strategies [13,14].

Convergent evidence has proposed that the processes related to formation and maintenance of the implicit habits mainly rely on the intact functioning of the striatum [15,16,17,18], while allocentric spatial learning [19,20] and the formation of cognitive maps [21] critically depend on intact functioning of the hippocampus. However, this distinction is probably an overgeneralization. In reality, the striatal and hippocampal regions appear to be not entirely segregated and both of them may contribute to navigational abilities regardless of the strategy carried out. In fact, both structures contain neurons whose firing is correlated with animal’s location, directional heading, some egocentric movements and behavioral trajectories, as well as neurons sensitive to changes in reward location [22]. Amidst these similarities, however, some different representational properties may promote a differential role of hippocampus and striatum during spatial learning and bias the significance of their behavioral output. Thus, similar spatial information may be processed and represented in more than one brain structure, and in particular hippocampal and striatal neurons may be sensitive to the same environmental manipulations to contribute to the final behavioral output, that is, the navigational strategy carried out [23].

Outstanding issues on this topic are what neuro-modulatory factors and how brain structures direct the selection of a navigational strategy from a wide repertoire of strategies. The functionality of Endocannabinoid System (ECS) in structures featured by high synaptic plasticity and related to associative learning and goal-directed behavior has made ECS a critical target for studies on learning and memory [24,25,26,27,28]. ECS is formed by cannabinoid receptors, their endogenous lipid ligands (endocannabinoids) and the machinery for synthesis and degradation of endocannabinoids [29,30]. Most central ECS functions are mediated by cannabinoid type-1 receptors (CB_1_) [31,32] that are densely expressed in neocortex, basal ganglia, amygdala, hippocampus, hypothalamus and cerebellum [30,31,32,33]. These receptors are principally found at presynaptic terminals and modulate the delivery of excitatory and inhibitory neurotransmitters, usually inhibiting their release. Demanding or stressful experiences trigger ECS activation in hippocampus [34,35], amygdala [36], striatum [37] and thalamus [38]. Systemic or intracerebral administrations of CB_1_ agonists and paradoxically antagonists impair the consolidation of spatial [39,40,41] and fear [26,42] memories. These controversial findings indicate complex ECS effects on memory that cannot be merely defined as impairing or enhancing effects of endocannabinoid activation and deactivation.

**Figure 1:**
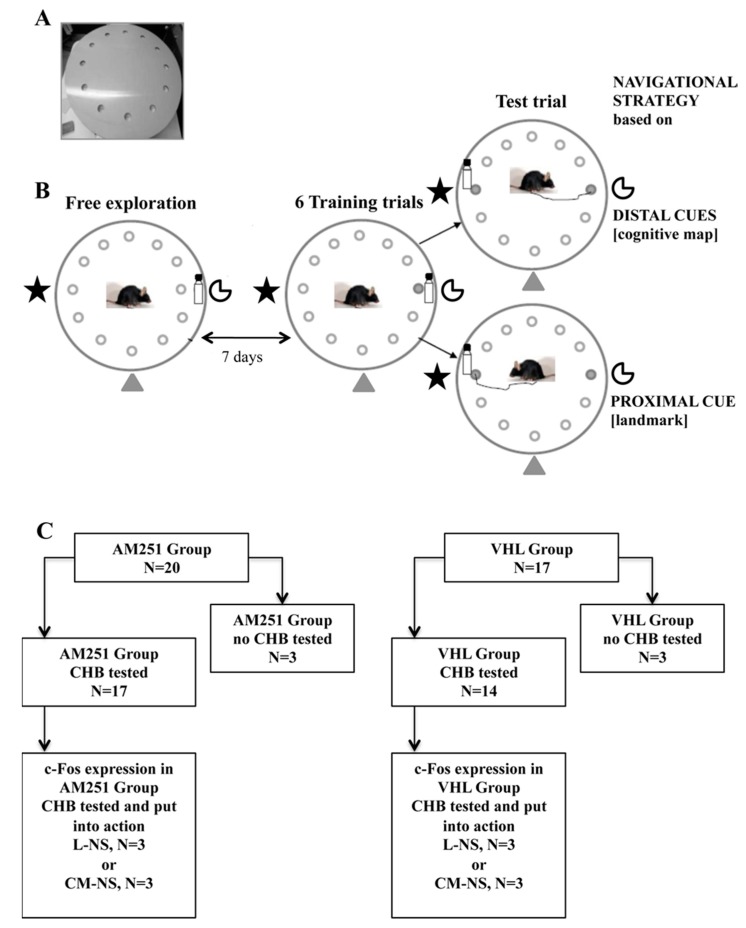
Circular Hole Board (CHB). (**A**) apparatus. (**B**) experimental procedures. (**C**) experimental groups: On the left, animals injected with AM251 (AM251 group, *n* = 20) and tested (*n* = 17) or not (*n* = 3) in the CHB. On the right, animals injected with vehicle (VHL group, *n* = 17) and tested (*n* = 3) or not (*n* = 3) in the CHB. To analyze c-Fos expression, we selected six animals (of which three belonging to the AM251 group and three to the VHL group) that used the Landmark-related Navigational Strategy (L-NS) in the CHB, and six animals (of which three belonging to the AM251 group and three to the VHL group) that used the Cognitive Map-related Navigational Strategy (CM-NS) in the CHB.

Starting from these remarks, the present study focuses on the involvement of ECS in the selection of navigational strategies related to landmark or cognitive mapping. To this aim, adult mice were treated with AM251 or with vehicle (VHL). Although exhibiting properties of inverse agonist at CB_1_ receptors, the AM251 may behave as a CB_1_ receptor antagonist according to its pharmaco-kinetic and -dynamic properties, in the various brain regions. Even if it is difficult to distinguish in vivo whether the AM251 acts as inverse agonist or antagonist at the CB_1_ receptors, it has to be underlined that both actions (as inverse agonist or as antagonist) are consistent in deactivating the ECS. Thus, in the present study the AM251 was used to reduce the ECS functionality acting on CB_1_ receptors during a navigational task. In order to achieve this aim, the treated animals were subjected to the Circular Hole Board (CHB) (Figure 1A,B), a task that can be solved by using different navigational strategies. Mice can escape from the maze either by remembering the spatial position of the exit hole, thus using the allocentric navigational strategy (CM-NS), or by following the proximal landmark, thus using the implicit associative learning (L-NS) (Figure 1B). At the end of the behavioral testing, the brain activation was evaluated by using cellular imaging of the activity-dependent protoncogene c-Fos in specific regions of the hippocampus (CA1, CA3 and Dentate gyrus—DG), dorsal striatum (dorsolateral striatum—DLS, dorsomedial striatum—DMS) and basolateral amygdala (BLA) (Figure 1C). 

The present study highlights the involvement of CB_1_ receptors as part of the selection system of the navigational strategies implemented to efficiently solve the spatial problem.

## 2. Results

### 2.1. CHB Behavioral Testing

#### 2.1.1. Free Exploration Trial

**Figure 2:**
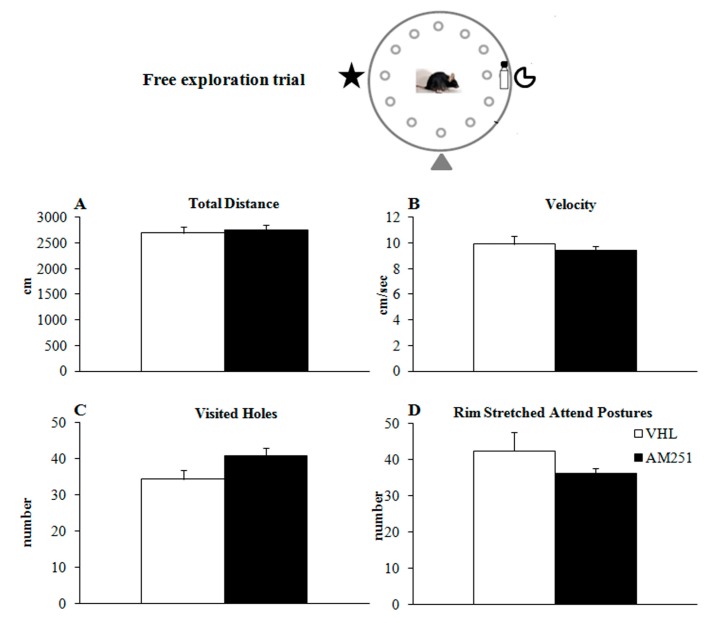
Behavior in the free exploration trial. Total distance (**A**), velocity (**B**), visited holes (the mouse put at least its nose in the hole) (**C**) and rim stretched attend postures (the mouse looked over the edge of the board) (**D**) exhibited by animals injected with AM251 or vehicle (VHL). The data presented as mean and standard errors were analyzed by one-way ANOVAs.

During the initial free exploration trial (Figure 1B), no differences in exploratory behavior of animals before treatment were found, as revealed by one-way ANOVAs on total distances (Figure 2A) (F_1,29_ = 0.20, *p* = 0.66), velocity (Figure 2B) (F_1,29_ = 0.62, *p* = 0.43), visited holes (Figure 2C) (F_1,29_ = 3.70, *p* = 0.07), rim stretched attend postures (Figure 2D) (F_1,29_ = 1.66, *p* = 0.21), grooming (F_1,29_ = 0.86, *p* = 0.36) and defecations (F_1,29_ = 0.53, *p* = 0.47).

#### 2.1.2. Training Trials

**Figure 3:**
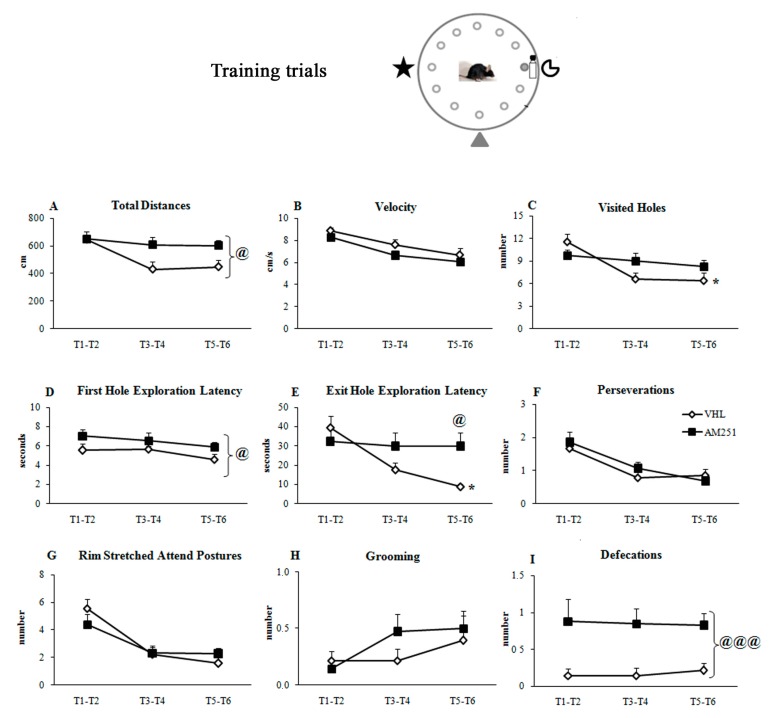
Behavior in the training trials. Total distances (**A**), velocity (**B**), visited holes (**C**), first hole exploration latency (**D**), exit hole exploration latency (**E**), perseverations (the mouse visited the same hole or at least two adjacent holes twice in a row) (**F**), rim stretched attend postures (**G**), grooming (**H**) and defecations (**I**) exhibited by animals injected with AM251 or vehicle (VHL). The data are presented as mean and standard errors. For each parameter, the values of trials 1-2 (T1-2), 3-4 (T3-4) and 5-6 (T5-6) were mediated and analyzed by two-way ANOVAs (group x trials). Significant *group* effect: @: *p* ≤ 0.05; @@@: *p* < 0.0005; significant Interaction: * *p* ≤ 0.05.

During the six training trials (Figure 1B), when the position of the exit hole was kept fixed with respect to the proximal and distal cues, AM251 group exhibited impaired spatial learning. Namely, AM251 animals travelled distances longer than VHL mice (Figure 3A) although they exhibited the same velocity (Figure 3B). Furthermore, while VHL group decreased the number of visited holes (Figure 3C) as trials went by, AM251 group maintained the same performance throughout the whole training. In comparison to VHL group, AM251 group showed longer latencies in exploring the first hole (Figure 3D) and reaching the exit hole (Figure 3E), and exhibited values not significantly changing throughout the whole training. While the number of perseverations (Figure 3F), rim stretched attend postures (Figure 3G) and grooming (Figure 3H) was similar between groups, the number of defecations of AM251 animals was higher than in VHL animals (Figure 3I). Statistical results of two-way ANOVAs on all parameters of the training trials are reported in Table 1.

#### 2.1.3. Test Trial

**Figure 4:**
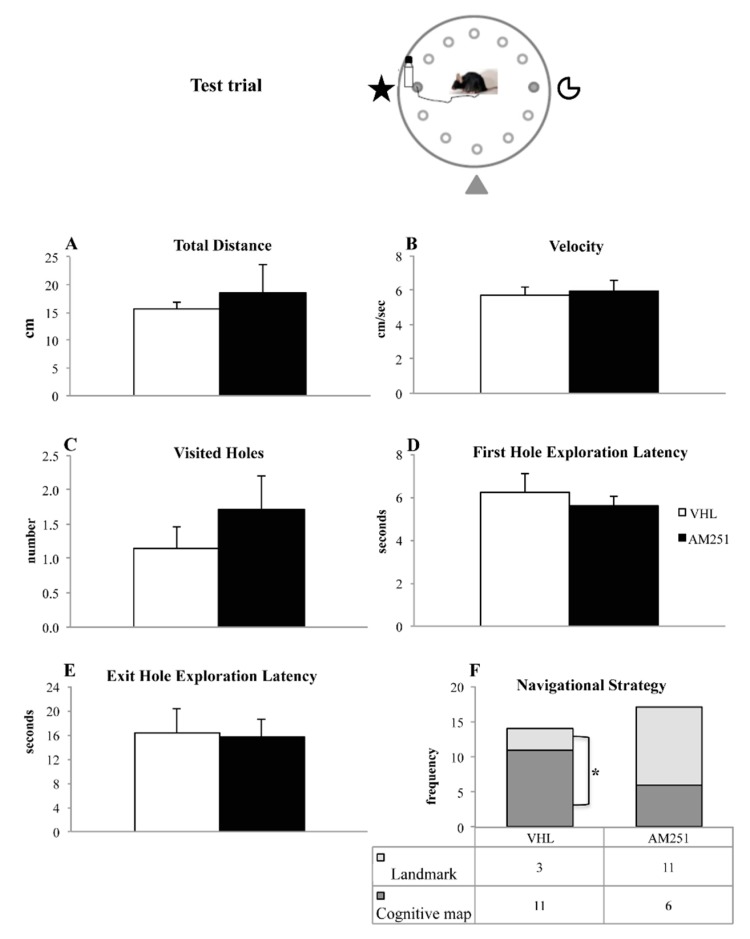
Behavior in the test trial. Total distances (**A**), velocity (**B**), visited holes (**C**), first hole exploration latency (**D**), exit hole exploration latency (**E**) and navigational strategy (**F**) exhibited by animals injected with AM251 or vehicle (VHL). For parameters **A**–**E**, the data were analyzed by one-way ANOVAs, while the frequencies of the parameter Navigational Strategy were compared by χ^2^ test. * *p* ≤ 0.05.

During the test trial (Figure 1B), the distal cues remained fixed with respect to the position of the exit hole, but the landmark (bottle) was relocated from hole 3 to hole 9. One-way ANOVAs on total distances (Figure 4A) (F_1,29_ = 1.92, *p* = 0.18), velocity (Figure 4B) (F_1,29_ = 0.23, *p* = 0.64), visited holes (Figure 4C) (F_1,29_ = 0.81, *p* = 0.37), first hole exploration latency (Figure 4D) (F_1,29_ = 0.45, *p* = 0.51) and exit hole exploration latency (Figure 4E) (F_1,29_ = 0.01, *p* = 0.90) failed to reveal any significant effect. More interestingly, significant association (χ^2^ = 5.80, *p* = 0.02) was found between AM251 treatment and navigational strategy put into action. In fact, in AM251 group the number of animals exhibiting the L-NS (*n* = 11) was not significantly different from the number of animals exhibiting the CM-NS (*n* = 6) (χ^2^ = 1.47, *p* = 0.23). Conversely, in VHL group the number of animals exhibiting the CM-NS (*n* = 11) was significantly higher than the number of animals exhibiting the L-NS (*n* = 3) (χ^2^ = 4.57, *p* = 0.03) (Figure 4F).

### 2.2. c-Fos Immunohistochemistry

**Figure 5:**
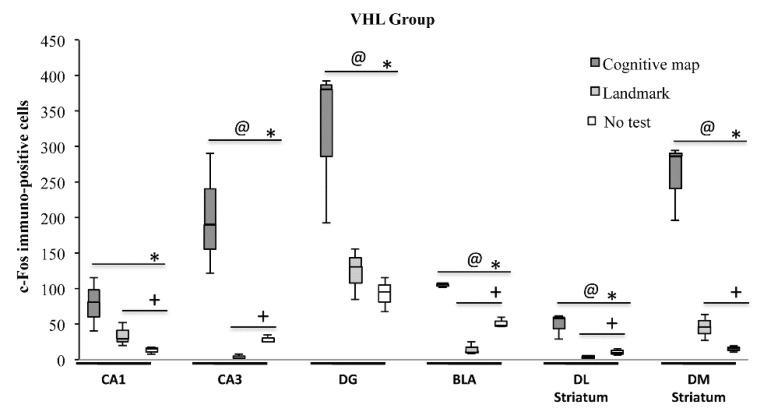
c-Fos activation in hippocampal CA1 and CA3 and dentate gyrus (DG), basolateral amygdala (BLA), dorsolateral (DL) and dorsomedial (DM) striatum exhibited by animals injected with vehicle (VHL) according to the landmark- or cognitive map-related navigational strategy carried out in CHB and in No test condition. Data were analyzed by Kruskal-Wallis test followed by Mann-Whitney U when appropriate. VHL animals which carried out the cognitive map-related navigational strategy showed a number of c-Fos^+^ cells significantly (@, *p* = 0.05) higher than the VHL animals which carried out landmark-related navigational strategy in all regions of interest but in CA1. Furthermore, VHL animals that carried out the cognitive map-related navigational strategy showed a number of c-Fos^+^ cells significantly (*, *p* = 0.05) higher than the not tested VHL animals in all regions analyzed. In comparison to not tested VHL animals, the VHL animals that carried out the landmark-related navigational strategy showed a number of c-Fos^+^ cells significantly (+, *p* = 0.05) higher in CA1 and DM Striatum, lower in CA3, BLA and DL Striatum and not statistically different in DG.

As regards the VHL group, significant differences in c-Fos expression were found among animals that put into action either navigational strategy and animals not behaviorally tested in all regions analyzed (CA1: H = 6.49, *p* = 0.03; CA3: H = 7.20, *p* = 0.03; DG: H = 5.95, *p* = 0.05; BLA, DLS and DMS: always H = 7.20, *p* = 0.02) (Figure 5). Interestingly, VHL animals which carried out the CM-NS showed a number of c-Fos^+^ cells significantly (always Z = 1.96, *p* = 0.05) higher than the VHL animals which carried out the L-NS in all regions of interest but in CA1 (H = 1.52, *p* = 0.13). Furthermore, VHL animals which carried out the CM-NS showed a number of c-Fos^+^ cells significantly (always Z = 1.96, *p* = 0.05) higher than the not tested VHL animals in all regions analyzed. In comparison to not tested VHL animals, the VHL animals which carried out the L-NS showed a number of c-Fos^+^ cells significantly (always Z = 1.96, *p* = 0.05) higher in CA1 and DMS, lower in CA3, BLA and DLS, and not statistically different in DG (H = 1.09, *p* = 0.27).

**Figure 6:**
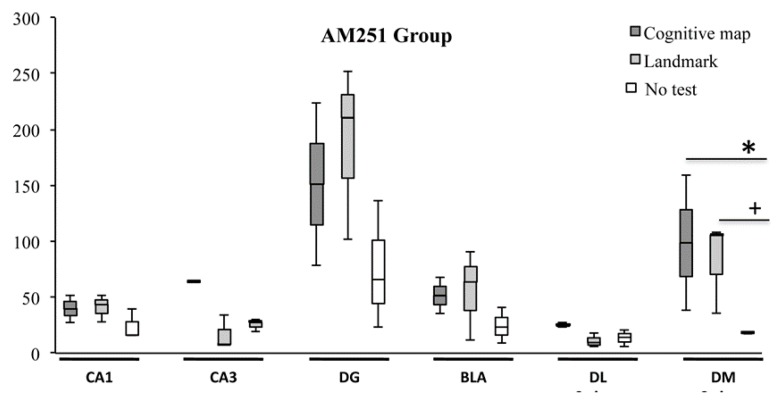
c-Fos activation in hippocampal CA1, CA3 and dentate gyrus (DG), basolateral amygdala (BLA) and dorsolateral (DL) and dorsomedial (DM) striatum exhibited by animals injected with AM251 according to the landmark- or cognitive map-related navigational strategy carried out in CHB and in No test condition. Data were analyzed by Kruskal-Wallis test followed by Mann-Whitney U when appropriate. In DM Striatum, AM251 animals that carried out the cognitive map-related navigational strategy expressed a number of c-Fos^+^ cells significantly (*, *p* = 0.05) higher than the not tested AM251 animals, while the AM251 animals that carried out the landmark-related navigational strategy expressed a number of c-Fos^+^ cells significantly (+, *p* = 0.05) higher than the not tested AM251 animals.

As regards the AM251 group, no significant differences in c-Fos expression were found in CA1 (H = 3.47, *p* = 0.18), CA3 (H = 5.60, *p* = 0.06), DG (H = 3.28, *p* = 0.19), BLA (H = 2.75, *p* = 0.25) and DLS (H = 5.60, *p* = 0.06), while a significant difference was found in DMS (H = 5.95, *p* = 0.05) (Figure 6). In this region, AM251 animals which carried out the CM-NS expressed a number of c-Fos^+^ cells not significantly different (Z = 1.09, *p* = 0.27) in comparison to AM251 animals which carried out the L-NS, but significantly (Z = 1.96, *p* = 0.05) higher than the not tested AM251 animals. Furthermore, AM251 animals which carried out the L-NS expressed a number of c-Fos^+^ cells significantly (Z = 1.96, *p* = 0.05) higher than the not tested AM251 animals.

**Figure 7:**
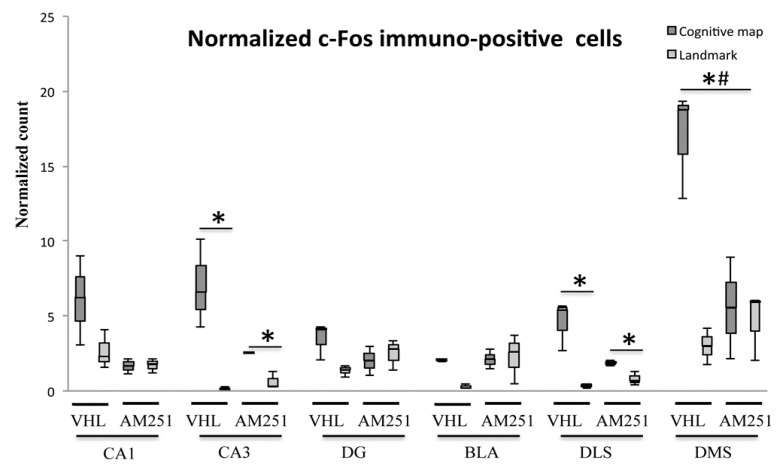
Comparison of normalized c-Fos activations in hippocampal CA1 and CA3 and dentate gyrus (DG), basolateral amygdala (BLA) and dorsolateral (DLS) and dorsomedial (DMS) striatum exhibited by animals injected with vehicle (VHL) or AM251 according to the landmark- or cognitive map-related navigational strategy carried out in CHB. Data were analyzed by Kruskal-Wallis test followed by Mann-Whitney U when appropriate. In CA3 and DLS, the VHL or AM251 animals that carried out the cognitive map-related navigational strategy expressed a normalized number of c-Fos^+^ cells significantly (*, *p* = 0.05) higher than the VHL or AM251 animals that carried out the landmark-related navigational strategy. In DMS, VHL (but not AM251) animals that carried out the cognitive map-related navigational strategy expressed a number of c-Fos^+^ cells significantly (*, *p* = 0.05) higher than the VHL animals that carried out the landmark-related navigational strategy, and higher (#, *p* = 0.05) than the AM251 animals using the cognitive map-related navigational strategy.

Additional analyses were carried out by computing the normalized c-Fos^+^ cell count per group. Significant differences were found in CA3 and DLS (always H = 9.05, *p* = 0.03) as well as in DMS (H = 7.51, *p=* 0.05), but not in CA1 (H = 4.18, *p* = 0.24), DG (H = 3.92, *p* = 0.27) and BLA (H = 6.69, *p* = 0.08) between animals exhibiting either navigational strategy (Figure 7). Namely, in CA3 and DLS the VHL or AM251 animals which carried out the CM-NS expressed a number of c-Fos^+^ cells significantly (Z = 1.96, *p* = 0.05) higher than the VHL or AM251 animals that carried out the L-NS. The results found in DMS revealed very interesting differences between groups: in fact, VHL (but not AM251) animals which carried out the CM-NS expressed a number of c-Fos^+^ cells significantly (always Z = 1.96, *p* = 0.05) higher than the VHL animals that carried out the L-NS, and higher than the AM251 animals using the CM-NS. 

Summing up: in VHL animals, the navigational strategy based on cognitive mapping determined a significant activation in all brain regions taken into account—the navigational strategy based on landmark determined a significant activation in CA1 and DMS, a significant de-activation in CA3, BLA and DLS, while no effect was found in DG in comparison to the no-test condition; AM251 treatment blocked the activation in DMS that characterized the navigational strategy based on cognitive mapping. 

## 3. Discussion

The present study investigated the role of ECS in selecting navigational strategies carried out by mice treated with AM251 or vehicle. We also analyzed the neuronal activation of hippocampus, dorsal striatum and amygdala according to the navigational strategy carried out. 

In the first CHB trial, which assessed animals’ free explorative behavior before CB_1_ pharmacological blockade, no significant differences were found on any variable between groups to be treated with AM251 or VHL.

Conversely, during the six training trials AM251 modulated spatial learning and emotional reactivity. Namely, when compared to VHL animals, AM251 animals showed longer distances, longer latencies in visiting the first hole and greater number of defecation boluses. Moreover, while the VHL group significantly decreased number of visited holes and exploration latencies to find the exit hole throughout all training trials, the AM251 group continued to show high values in these parameters, without displaying any learning effect. 

Various studies highlighted ECS involvement in spatial learning and memory [43,44,45,46]. Namely, it has been described that CB_1_ receptor knock-out mice and wild-type mice exhibited identical rate of acquisition of platform position in the Morris Water Maze (MWM) when the hidden platform was kept fixed; vice versa, in a reversal task in which the location of the hidden platform was moved in a different position, the mutant mice continued to visit the original platform location. Furthermore, CB_1_ functionality affects spatial working memory and facilitates extinction and/or forgetting processes as indicated by (endo)cannabinoid agonists administration [26,47]. It has been reported that the CB_1_ antagonist Rimonabant, when systemically administered, impaired MWM spatial learning, by increasing swimming speed and thigmotaxis, while when intrahippocampally infused, it facilitated learning ability, by shortening path lengths to locate the platform, without affecting long-term consolidation of spatial memory [48]. The authors have interpreted the different effects between administration regimes in terms of CB_1_ blockade in non-memory- and memory-related brain regions, advancing a memory enhancing effect linked to the selective inactivation of hippocampal CB_1_ receptors [48]. However, since the effects of endocannabinoids on memory are strongly dependent on more or less aversive context and on stress levels during training [49], in evaluating the effects of CB_1_ blockade on spatial learning the emotional load of the task has to be taken into account. A sign of enhanced emotional reactivity of the AM251 animals could be represented by the significantly higher number of defecation boluses in comparison to VHL animals. However, it should be noted that the ECS controls several aspects of gastrointestinal functions, independently from emotional load. Namely, CB_1_ antagonists activate gastrointestinal motility, peristalsis, defecation and secretions in a dose-dependent manner, while CB_1_ agonists inhibit these effects [50]. 

During the test phase, in which the exit hole position remained constant with respect to the distal cues but shifted with respect to the landmark, the two experimental groups differently put the two spatial strategies into action. Namely, while most VHL animals revealed as cognitive map-learners, AM251 animals carried out either navigational strategy without any preference. Notably, VHL group that exhibited an efficient learning during the training phase displayed a dominance of cognitive map-related strategy at the expense of landmark-related strategy. This prominence was not present in AM251 group that not by chance exhibited an impaired learning during the training phase. 

Interestingly, the navigational strategy each animal used was closely associated to the profile of c-Fos expression in specific brain areas. In VHL animals, the predominant strategy based on cognitive mapping was associated with the neuronal activation in the entire hippocampus (CA1, CA3 and DG), amygdala (BLA) and dorsal striatum (DLS and DMS), while the less frequently used strategy based on landmark was associated with the activation in CA1 and DMS and de-activation in CA3, BLA and DLS. Furthermore, in comparison to L-NS c-Fos expression profile also evidenced the distinct contribution of hippocampal DG as well as the elevated activation of DMS in CM-NS. Thus, on one hand the two navigational strategies share the parallel activation of some hippocampal and dorso-striatal areas, and on the other hand they are characterized by distinct neuronal patterns. The present data are in line with those reported by Fouquet et al. [51] as well as with the previously advanced conceptualization of the organization of the memory systems. This vision assumes that many, but not all, hippocampal and striatal regions are continuously engaged in similar, though not identical, context-dependent tasks [22]. Specifically, the striatum and hippocampus co-operate to implement the appropriate behavioral output in front of new environmental requests, as after a change in visual context, regardless of whether the task is solved through a flexible cognitive mapping or a landmark-directed strategy. This latter strategy differs from the bare egocentric strategies that represent the object location in space relative to the body axes, as well as from the sequential or serial strategies that require a temporal order memory of successive choice points [51] or the unidirectional exploration of adjacent points [41], respectively. In fact, the landmark-based strategy is centered on a learned association between a well-defined, current external stimulus and a behavior to achieve the goal, without exploiting any spatial competence. As a result, such a strategy is relatively inflexible and slow to acquire because its processing does not take advantage of the more rapid learning feasible when outcomes are driven by the spatial cognitive mapping. 

Interestingly, we found that the strength of influence of the neuronal structures on behavioral output was modulated by endocannabinoid functioning. To study such a modulation, here, we administrated AM251. As previously described, this drug works as an inverse agonist or antagonist at CB_1_ receptors. The inverse agonism may be explained considering the two-state model of receptors, which proposes that they can be constitutively active (“on” state: they are coupled to their effector mechanisms even in the absence of agonists) or inactive (“off” state: they are not spontaneously coupled to their effector mechanisms). Agonists increase the proportion of receptors in the “on” state, inverse agonists increase it in the “off” state, and antagonists leave the number of receptors in each state unaffected. In detail, while the agonists are featured by both affinity (binding to the target receptor) and intrinsic efficacy (changing receptor activity to produce a response) and the antagonists are featured by affinity but no intrinsic efficacy (binding to the target receptor without producing any response), the inverse agonists have negative intrinsic efficacy (decreasing the activity of a receptor) [52]. When there the constitutive receptor activity and the action of an endogenous agonist are present, as occurs in the case of ECS, an antagonist will reduce the component of the response due to the endogenous agonist, and an inverse agonist will reduce both the endogenous agonist component and constitutive receptor activity. Therefore, the effect of the inverse agonist will be consistent with, but greater than, that of the antagonist. Notably, it has to be taken into account that AM251 that exhibits properties of inverse agonist may behave as an antagonist in different brain regions depending on dosage, its intrinsic efficacy and the magnitude of constitutive receptor activity specific in the various cell phenotypes. In fact, there is evidence that AM251 may act as CB_1_ receptor antagonist at low concentrations and as inverse agonist at high concentrations [53,54]. In the present study AM251 was used at a dose of 1 mg/kg i.p., a dose that is controversially retained either evoke an inverse agonist response or block CB_1_ receptors and prevent the effects of CB_1_ agonists [55,56,57,58,59]. In short, it is challenging to discriminate in vivo the AM251 action as inverse agonist at the CB_1_ receptor or antagonist blocking endogenous endocannabinoids acting at a constitutively active CB_1_ receptor. 

As an additional complexity in facing ECS, it has to be taken into account that AM251 exhibits significant activity not only at CB_1_ as an inverse agonist/antagonist, but also at orphan cannabinoid G-protein-coupled receptor 55 (GPR55) as an agonist [60,61,62,63]. The lack of homology between cannabinoid receptors may explain the differences among CB_1_ and GPR55 in the signaling system and downstream cascade. While the CB_1_ receptor is coupled to inhibitory G proteins (which inhibit the adenylyl cyclase and increase the activation of the mitogen-activated protein kinase), and regulates the activity of Ca^2+^ and K^+^ channels, and consequently, inhibits neurotransmitters release, the GPR55 promotes the outflow of calcium from intracellular stores via phospholipase C activation, and accordingly, facilitates neurotransmitters release. 

Studies in rodent showed that GPR55 is present in hippocampus, thalamus, forebrain, frontal cortex, hypothalamus, cerebellum and striatum [64]. There is increasing evidence of co-expression and cross-talk between cannabinoid receptors supporting the notion that ligands binding to the CB_1_ receptor could influence the response to ligands acting through GPR55, and vice versa [65]. In this framework, under the action of AM251, on the one hand the activity of inhibitory G proteins coupled to CB_1_ receptors is inhibited, and consequently, the neurotransmitter release is allowed; on the other hand, the function of G proteins coupled to GPR55 is activated and consequently the neurotransmitter release is facilitated. As a result, the opposite signaling between CB_1_ and GPR55 leads to accordant behavioral consequences. We cannot exclude that some behavioral and immunochemical effects observed in the present research may involve GPR55 instead or additionally to the classic activity of CB_1_; however, the detailed examination of the respective roles goes beyond the scope of the present work.

Recently, the functionality of GPR55 in the procedural memory has been investigated. Rats bilaterally injected into the dorsolateral striatum with a GPR55 and CB_1_ agonist and simultaneously with AM251 showed the most efficient performance in a procedural memory task [66]. To further investigate the GPR55 role in spatial learning, rats that received bilateral infusions of a GPR55 agonist into the hippocampus exhibited serial strategy at the expense of spatial strategy, suggesting that integrity of GPR55 pathway could be required to establish a specific navigational strategy [67]. Accordingly, injections of palmitoylethanolamide into the ventral hippocampus affected spatial memory, probably via GPR55 [68], and hippocampal GPR55 stimulation improved synaptic plasticity [69].

Coming back to the more classic literature on CB_1_ that underlines how the selection of navigational strategies is biased by manipulating CB_1_ functionality, previous findings describe that during the light phase of the diurnal cycle the AM251 intra-hippocampal administration results in a decrease of the spatial strategy, while AM251 intra-striatal administration results in an increase in random strategy [41]. Not surprisingly, in the hippocampus but not in the striatum, CB_1_ circadian variations were present [41]. Furthermore, systemic and local administrations of drugs acting on ECS affect the consolidation of spatial and emotional memories without altering memory and time of exploration in Novel Object Recognition test [55,70]. 

In the present study, the CB_1_ blockade elicited by AM251 treatment markedly reduced DMS activation that characterizes the strategy based on cognitive mapping, and at same time reduced the numbers of animals putting this strategy into action. DMS is involved in processes mediating goal-directed behavior or decision-making, such as action-outcome associations, behavioral flexibility and action selection [71,72]. Namely, DMS integrates sensory, cognitive and value-based information, along with motor feedback to form the complex associations that control action selection and goal-directed behaviors; furthermore, it links the goal with more suitable spatial behavior [73]. These operations are required for the acquisition of new strategies and for the flexible re-adjustment of already present strategies when the environment changes [72]. Much of the evidence that DMS is the site of plasticity for these behaviors derives from the observations that disrupting specific inputs to DMS impairs goal-directed learning [74] and that DMS contains reward-responsive, stimulus-related and location-related neurons [75,76,77]. From a computational point of view, it has been formalized the DMS role in learning and expression of navigational strategies, no matter if the used internal model is allocentric or egocentric [78]. Thus, DMS acts as a transition point in which learning is translated into action and behavioral flexibility is allowed [72,79,80]. 

At the cellular level, DMS is composed primarily of 95% GABAergic medium spiny neurons (MSN) that integrate cortically processed information about the environment, internal body state and past experience, to select actions that maximize positive and minimize negative outcomes. The different portions of MSN dendrites receive synaptic connections from distinct brain areas: in particular, inputs to the cell body and proximal dendrites derive from GABAergic and cholinergic striatal interneurons, while inputs to distal dendrites derive from glutamatergic cortical, dopaminergic nigrostriatal and thalamic afferents [81]. Recently, the plastic structural changes that cannabinoid administration elicits on striatal MSN have been investigated: treatment with THC, a CB_1_ partial agonist, selectively increased dendritic spine density in distal dendrites of MSN belonging to DMS, whereas no effects were observed in DLS neurons. This observation fits the effects of AM251 treatment on DMS found in the present research, and makes us consider DMS as the crucial structure that may provide information about the role of CB_1_ in the selection between navigational strategies. In fact, the multi-pronged observations that the manipulations on CB_1_ activity modified DMS spine density [82], brought about changes in navigational behaviors (present findings, [41]), modulated c-Fos activation in DMS (present findings), are related to the presence of presynaptic CB_1_ receptors (which is known to inhibit the neurotransmitter release) in both cortical glutamatergic afferents on and GABAergic terminals in MSN and in aspiny interneurons of dorsal striatum [83,84]. Furthermore, the present data indicate that to select more frequently the navigational strategy related to cognitive map it is required the full functionality of DMS neurons. When AM251 blocks CB_1_ activity and consequently either navigational strategy can be selected without any preference, GABA release by DMS is favored either by a direct dis-inhibition of MSN and aspiny interneurons and by an indirect facilitation of the cortical glutamatergic afferents impinging on MSN [25]. As a result, a downstream inhibition is promoted, in line with the general reduced c-Fos activation observed in the present research. The observation that rats with a unilateral cortico-DMS disconnection are still capable of acquiring goal-directed actions, while rats with bilateral disconnection are severely impaired [74] represents further evidence to support the present finding that different activation levels in a specific structure are related to different behavioral outcomes. 

## 4. Material and Methods

### 4.1. Subjects

Male adult (2.5 month-old) C57BL/6JOlaHsd mice (Envigo, Udine, Italy) were used in the present research. The animals were group-housed (four mice/cage) with food (Mucedola, Milan, Italy) and water *ad libitum*, and kept under a 12-h light/dark cycle with light on at 07:00 h, controlled temperature (22–23 °C) and constant humidity (60 ± 5%). All experiments took place during the light phase. All efforts were made to minimize animal suffering and to reduce their number, in accordance with the European Directive (Directive 2010/63/EU, 22/09/2010). 

Mice were randomly assigned to: AM251 group encompassing animals (*n* = 17) injected with AM251 and then tested in the CHB; VHL group encompassing animals (*n* = 14) injected with VHL and then tested in the CHB. For c-Fos quantification we selected six animals (of which three belonging to the AM251 group and three to the VHL group) that in used the L-NS in the CHB, and six animals (of which three belonging to the AM251 group and three to the VHL group) that in used the CM-NS in the CHB. Furthermore, to compare neuronal activation in the absence of any behavioral testing we performed c-Fos immunostaining in a further six mice injected with AM251 (*n* = 3) or VHL (*n* = 3) without being behaviorally tested (Figure 1C).

### 4.2. Drugs

The animals treated with the drug acting on ECS (*n* = 20) were intraperitoneally (i.p.) injected with the AM251 (1 mg/kg; Tocris, Bristol, UK) dissolved in the VHL composed of saline solution with 10% DMSO and 5% Tween 80 and administered at volume of 5 mL/kg of body weight. The VHL animals (*n* = 17) received the same volume of VHL i.p.

The selection of AM251 dosage at 1 mg/kg was based on behavioral results on locomotor- [85] anxiety- [86] and reward- [87] related effects. The same dosage was used by Maione et al. [56] to investigate AM251 neurochemical properties.

### 4.3. CHB Testing

#### 4.3.1. Apparatus

The CHB is a revolving plexiglas grey round plate (110 cm in diameter; situated 1 m above the floor) with 12 holes at equal distances from each other, located 10 cm from the rim of the board. The holes are 5 cm in diameter and can be closed by a lid at a depth of 5 cm (Figure 1A). Whether a hole is open or closed can only be detected by the mouse putting its head over the edge of the hole. The holes were virtually numbered in a clockwise direction, considering holes like hours on a clock.

A ladder (5 cm width, 15 cm length) leads from the open exit hole down to a cage containing the sawdust from the animal’s cage. The proximal intra-maze cue was represented by a glass water bottle (50 cl) positioned near the exit hole, while the distal extra-maze cues allowing the spatial orientation on the board were provided by multiple colorful and geometric shapes on the room walls kept fixed throughout the experiment.

Behavior was digitally recorded and analyzed with Ethovision XT (Noldus, Wageningen, The Netherlands) that sampled the position of the mouse 12.5 times per second. Experimenters who performed the behavioral testing were blind to the drug treatment.

#### 4.3.2. Procedures

Each trial started by placing the mouse in a cylinder (Plexiglas; 25 cm height; 10 cm diameter) located at CHB center. After 5 s, the cylinder was lifted and the mouse could explore the board and exit through the open tunnel. If the mouse did not find the exit hole within 120 s, it was gently guided to the exit using a grid. After each trial the board was cleaned with 30% alcohol to dissipate odor cues. 

Five days before behavioral experiments started, the mice were trained to descend the ladder to reach the home cage for three consecutive days (10 min/day).

#### 4.3.3. Free Exploration Trial

To evaluate the general locomotor activity before the pharmacological treatment in the first trial (free exploration trial) (Figure 1B) the mice were allowed exploring the CHB for 5 min. In this first trial, all holes were closed, the intra-maze landmark (proximal cue: glass bottle, 13 cm height) was positioned near hole 3 that would become the exit hole in the subsequent trials, but which was still closed; multiple extra-maze distal cues were located on room walls.

The following parameters were calculated: total distances (cm), velocity (cm/s), visited holes, when the mouse put at least its nose in the hole, rim stretched attend postures, when the mouse looked over the edge of the board, grooming and defecations. At the end of the 5 min of exploration, hole 3 was opened and the mouse was guided there by the experimenter to get the ladder down for three consecutive times. 

#### 4.3.4. Training Trials

One week after the free exploration trial, mice were pharmacologically treated with AM251 or VHL. After 30 min, mice were given six successive 120 s-training trials with inter-trial interval of 15 min. The proximal landmark was positioned near the exit hole (hole 3) that was the only hole opened while the multiple distal cues were kept fixed on room walls. Thus, the location of the exit hole was always fixed relatively to intra- and extra-maze cues, allowing the animal to learn its location by taking into account the spatial relationships among distal extra-maze spatial cues as well as the association between proximal intra-maze cue and exit hole (Figure 1B). It should be noted that the proximal intra-maze cue was the preferential stimulus to build the L-NS (implicit associative learning), while the distal extra-maze cues were the appropriate stimuli to develop the CM-NS (spatial allocentric learning).

The following parameters were calculated: total distances (cm), velocity (cm/s), visited holes, first hole exploration latency (s), exit hole exploration latency (s), perseverations, when the mouse visited the same hole or at least two adjacent holes twice in a row, rim stretched attend postures, grooming and defecations. For each parameter, the values of the trials 1–2, 3–4 and 5–6 were mediated. 

#### 4.3.5. Test Trial

After 15 min, the test trial started (Figure 1B), in which distal extra-maze cues were kept fixed and the proximal intra-maze cue was relocated in the position opposite to its location during the training trials (from hole 3 to hole 9). Thus, hole 3 remained opened, and the “new exit” through hole 9 was filled with sawdust from the animal’s cage. Test trial allowed analyzing whether the navigational strategy used to solve the spatial task was based mainly on cognitive map or landmark. 

The following parameters were calculated: total distances (cm), velocity (cm/s), visited holes, first hole exploration latency (s), exit hole exploration latency (s) and navigational strategy (frequency) based on cognitive map or landmark.

### 4.4. Biochemical Analyses

#### 4.4.1. Tissue Preparation

Mice tested on CHB and mice not behaviorally tested were isolated for 1 h to perform c-Fos immunohistochemistry (modified from Conversi et al. [88]). Mice were deeply anesthetized and sacrificed by decapitation. The brains were removed, immersed overnight in paraformaldehyde 4% (PAF), cryoprotected with 30% sucrose solution, cut in serial 40 μm coronal sections with a freezing microtome and alternatively processed for c-Fos immunohistochemistry and Nissl staining. According to the mice stereotaxic atlas [89] by using Nissl sections as anatomical reference, the following regions of interest were identified: hippocampus (from −1.34 to −1.94 mm in relation to bregma), amygdala (from −1.34 to −1.94 mm), and dorsal striatum (from 1.18 to 0.62 mm).

#### 4.4.2. c-Fos Immunohistochemistry

Free floating coronal sections were washed three times in Phosphate-Buffer (PB) + 0.3% Triton X-100 (PBTX), incubated for 30 min in 0.3% H_2_O_2_ in PBTX to prevent endogenous peroxidase activity, washed three times in PBTX, incubated for 30 min in PBTX containing Avidin blocking solution (Vectastain Elite ABC Kit, Vector Laboratories, Peterborough, UK) (two drops/5 mL), washed three times in PBTX, incubated for 30 min in PBTX containing Biotin blocking solution (Vectastain Elite ABC Kit) (two drops/5 mL), washed again three times in PBTX and incubated overnight with primary antibody (Anti-c-Fos antibody ab 190289, AbCam, Cambridge, UK) diluted 1:5000 in PBTX + 5% normal goat serum. After three washes in PBTX, the sections were incubated for 2 h in PBTX containing secondary antibody (1:200, biotinylated Goat anti-Rabbit, Vectastain Elite ABC Kit), washed again three times in PBTX, incubated for 1 h in Avidin-Biotin complex (Vectastain Elite ABC Kit) diluted 1:50 in PBTX, washed three times in PB 0.1 M and then visualized with diaminobenzidine, as chromogen (DAB, ScyTek Laboratories, Logan, UT, USA) diluted 1:30. Finally, the sections were washed three times in PB 0.1 M, dehydrated in ethanol, cleared in xylene and coverslipped. 

We confirmed the specificity of the immunohistochemical pattern by omitting the primary antibody. Such a negative control resulted in the absence of c-Fos immunoreactivity in all brain regions.

#### 4.4.3. Cell Counting 

The quantitative analysis of the immunohistochemical reaction for c-Fos immuno-positive (^+^) cells was performed by doing a cell count along the coronal sections containing the regions of interest by using an optical microscope (Zeiss AxioLab, Oberkochen, Germany) integrated with an image acquisition system (DEI-750 Camera, Optronics, Goleta, CA, USA). For each structure of each subject, at least three sections were identified. 

**Figure 8:**
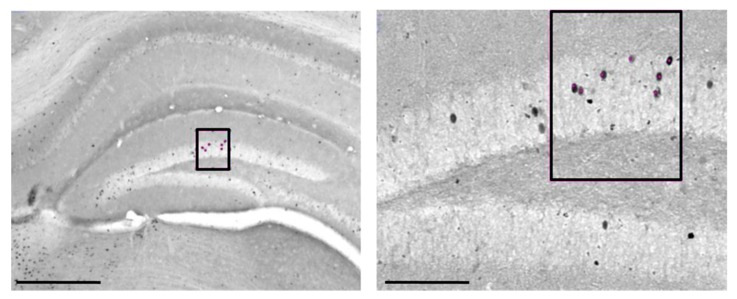
Representative photomicrographs of c-Fos+ cells (highlighted in the black boxes) in hippocampal CA1, CA3 and dentate gyrus (DG) of an animal treated with vehicle at lower (4×) (on the left, Scale bar: 1000 μm) and higher (20×) (on the right, Scale Bar: 200 μm) magnification.

Immunoreactive cells were counted in hippocampal CA1 and CA3, and DG (Figure 8), DMS and DLS, as well as BLA. The boundaries of the structures of interest were recognized with the help of the stereotaxic atlas [89] and the corresponding Nissl slide. For the cell counting, 4× images were used as TIFF files, in which the light and contrast levels were kept stable. c-Fos^+^ cell counting was performed using the public domain software ImageJ (http://rsb.info.nih.gov/ij) and in particular using the “Image-based Tool for Counting Nuclei” plugin (ITCN) to count active cores. The photomicrographs were converted into 8-bit images. The levels of “width”, “minimum distance” and “threshold” were also changed until the automated count was comparable to the same count made manually. The number of c-Fos^+^ cells was computed and compared within the AM251 groups (animals using L-NS or CM-NS as well as no tested animals) and VHL groups (animals using L-NS or CM-NS as well as no tested animals) (Figure 1C). Furthermore, additional analyses were carried out by calculating the mean count in each structure for each animal divided by the mean count in that region of the respective not tested animals to generate a normalized count for each animal. 

### 4.5. Statistical Analysis

Behavioral data (mean ± SEM) were firstly tested for normality (Wilk-Shapiro’s test) and homoscedasticity (Levene’s test) and then compared by one- and two-way ANOVAs, followed by Newman-Keuls test when appropriate. Frequencies of navigational strategies were compared by χ^2^ test. Since immunohistochemical data did not meet parametric assumptions, non-parametric analyses of variance (Kruskal-Wallis test, Mann-Whitney U) were used. All analyses were performed by using Statistica 7.0 for Windows (TIBCO Software Inc., Aliso Viejo, CA, USA), and differences were considered significant at *p* ≤ 0.05.

## 5. Conclusions

Our data show that the information coding only in the hippocampus is not sufficient to bias towards strategy based on cognitive mapping, and the information coding only in the DMS is not sufficient to drive strategy based on landmark. In fact, both structures continuously cooperate to perform successfully either allocentric or implicit associative learning. In this enlarged and concerted network, the prefrontal cortex may represent the hub modulating balance between the striatal and hippocampal joint activity since no direct anatomical connectivity is known [90]. 

Assuming that representations that persist across context changes reflect learned information, we make the following conclusions. A parallel processing occurs within hippocampus and striatum regardless of the navigational strategy selected, although the different hippocampal and striatal sub-regions effectively compete for control of behavioral outcome. The strength of the influence of the neural systems on the behavioral output is modulated by the CB_1_ receptors, prompting them to use different spatial navigational strategies to cope with the environmental demands.

## Figures and Tables

**Table 1:** Statistical results of two-way ANOVAs on the behavioral parameters of the training trials. In bold * are reported significant results.

Effect (Freedom Degrees)	Total Distances	Velocity	Visited Holes	First Hole Latency	Exit Hole Latency	Perseverations	Rim Stretched Attend Postures	Grooming	Defecations
Group F(1,29) *p*	4.01 0.05 *	2.42 0.13	0.80 0.37	6.27 0.02 *	2.42 0.13	1.16 0.69	0.05 0.83	0.66 0.42	16.29 0.0004 *
Trials F(2,29) *p*	6.40 0.003 *	25.67 ≤0.0001 *	10.50 0.0001 *	1.65 0.20	5.20 0.008 *	14.42 ≤0.0001 *	21.17 ≤0.0001 *	1.92 0.15	0.01 0.99
Interaction F(2,58) *p*	2.63 0.08	0.26 0.77	4.20 0.02 *	0.11 0.89	3.70 0.03 *	0.71 0.49	1.64 0.20	0.70 0.50	0.07 0.93
